# In vivo T1 mapping for quantifying glymphatic system transport and cervical lymph node drainage

**DOI:** 10.1038/s41598-020-71582-x

**Published:** 2020-09-03

**Authors:** Yuechuan Xue, Xiaodan Liu, Sunil Koundal, Stefan Constantinou, Feng Dai, Laura Santambrogio, Hedok Lee, Helene Benveniste

**Affiliations:** 1 Department of Anesthesiology, Yale School of Medicine, 330 Cedar St, TMP 3, New Haven, CT 06520 USA; 2 Department of Critical Care Medicine, Xiangya Hospital, Central South University, Changsha, 410008 Hunan China; 3 Yale Center for Analytical Sciences, Yale School of Public Health, New Haven, CT USA; 4 Englander Institute of Precision Medicine, Department of Radiation Oncology, Physiology and Biophysics, Weill Cornell Medicine, New York, NY 10065 USA

**Keywords:** Imaging, Biological techniques, Medical research, Preclinical research

## Abstract

Dynamic contrast-enhanced magnetic resonance imaging (MRI) for tracking glymphatic system transport with paramagnetic contrast such as gadoteric acid (Gd-DOTA) administration into cerebrospinal fluid (CSF) requires pre-contrast data for proper quantification. Here we introduce an alternative approach for glymphatic system quantification in the mouse brain via T1 mapping which also captures drainage of Gd-DOTA to the cervical lymph nodes. The Gd-DOTA injection into CSF was performed on the bench after which the mice underwent T1 mapping using a 3D spoiled gradient echo sequence on a 9.4 T MRI. In Ketamine/Xylazine (KX) anesthetized mice, glymphatic transport and drainage of Gd-DOTA to submandibular and deep cervical lymph nodes was demonstrated as 25–50% T1 reductions in comparison to control mice receiving CSF saline. To further validate the T1 mapping approach we also verified increased glymphatic transport of Gd-DOTA transport in mice anesthetized with KX in comparison with ISO. The novel T1 mapping method allows for quantification of glymphatic transport as well as drainage to the deep and superficial cervical lymph nodes. The ability to measure glymphatic transport and cervical lymph node drainage in the same animal longitudinally is advantageous and time efficient and the coupling between the two systems can be studied and translated to human studies.

## Introduction

The glymphatic system (GS) in rodents was first described by Iliff and Nedergaard in 2012 as a brain-wide perivascular transit passageway for cerebrospinal fluid (CSF) facilitating waste clearance in an aquaporin 4 (AQP4) water channel dependent manner^1^. Visualization and quantification of GS transport in whole mouse brain was performed ex vivo by first allowing tracers administered into CSF to circulate for a pre-fixed time interval (30 min–2 h) after which the rodents were euthanized and their brains examined for tracer uptake using fluorescence microscopy^1^. In 2013, we introduced dynamic contrast enhanced magnetic resonance imaging (DCE-MRI) as an in vivo method for tracking GS transport in the whole rodent brain^2^ and later refined gadolinium based contrast administration into CSF and GS quantification^3,4^. The DCE-MRI approach for brain-wide GS transport assessment has been widely used and adapted to different species including mice^5–7^, non-human primates^8^, and humans^9,10^. Recently, the DCE-MRI approach for GS transport assessment was extended to studies in awake rats.

The DCE-MRI GS method involves administering small molecular weight (MW) (e.g. gadopentetic acid MW 547 Da, gadoteric acid (Gd-DOTA), MW 559 Da) or large MW (e.g. GadoSpin P, MW 200 kDa) paramagnetic tracers into CSF via the cisterna magna (CM)^2,3^, cerebral lateral ventricles^11^ or lumbar intrathecal route^9^. Transport of the paramagnetic contrast molecule (a.k.a. ‘solute’) from CSF into brain parenchyma is tracked dynamically by a series of 3D T1-weighted MRI scans^1^. The time series of 3D T1-weighted spoiled gradient echo (SPGR) brain images acquired before, during and after solute delivery into CSF are used to quantify an ‘enhancement ratio’, (defined by % signal change from the baseline) used for GS transport assessment^2,12^. GS transport parameters are subsequently derived using computational processing including ‘time-signal curves’^2,5,13^, cluster analysis^2,12^, kinetic modelling^4,13^ or the optimal mass transport formulation^14–16^. To calculate the actual solute mass entering into the GS at the voxel-level we previously implemented a variable flip angle 3D spoiled gradient echo (VFA-SPGR) T1 mapping technique^3^. Using rats with indwelling CSF catheters positioned inside the MRI, we acquired 3D T1 whole brain maps dynamically before, during and several hours after Gd-DOTA CSF administration^2–4,16^. We subsequently demonstrated that GS transport kinetics including solute influx and efflux rate constants could be calculated in the whole rat brain from the time trajectories of Gd-DOTA concentrations extracted from CSF and brain tissue compartments^13^.

Here we report that the T1 mapping approach can also be used to measure GS transport in the mouse brain but in a more time-efficient and technically simplified manner not requiring indwelling CSF catheters during the MRI acquisitions. Specifically, the T1 mapping technique can be implemented as a single ‘snapshot’ paradigm timed from the Gd-DOTA CSF administration performed outside the MRI, which simplifies the experimental set-up related to MRI scanning. The T1 mapping method is also advantageous because it allows for GS transport quantification at the voxel-level in whole mouse brain and in addition captures solute drainage directly to the cervical lymph nodes (superficial as well as deep cervical lymph nodes) in the same animal. Here we introduce the novel T1 mapping approach and further validate it by implementing two anesthetic regimens known to alter brain-wide GS solute transport differently^17^. Specifically, we tested the following hypotheses: (1) GS solute transport can be quantified directly by T1 mapping in the whole mouse brain, (2) T1 mapping can capture drainage to cervical lymph nodes and 3) GS solute transport by T1 mapping will be increased in mice anesthetized with ketamine/xylazine (KX) in comparison to mice anesthetized with isoflurane (ISO) only.

## Results

### Experimental overview of T1 mapping for quantification of GS transport and drainage

**Figure 1:**
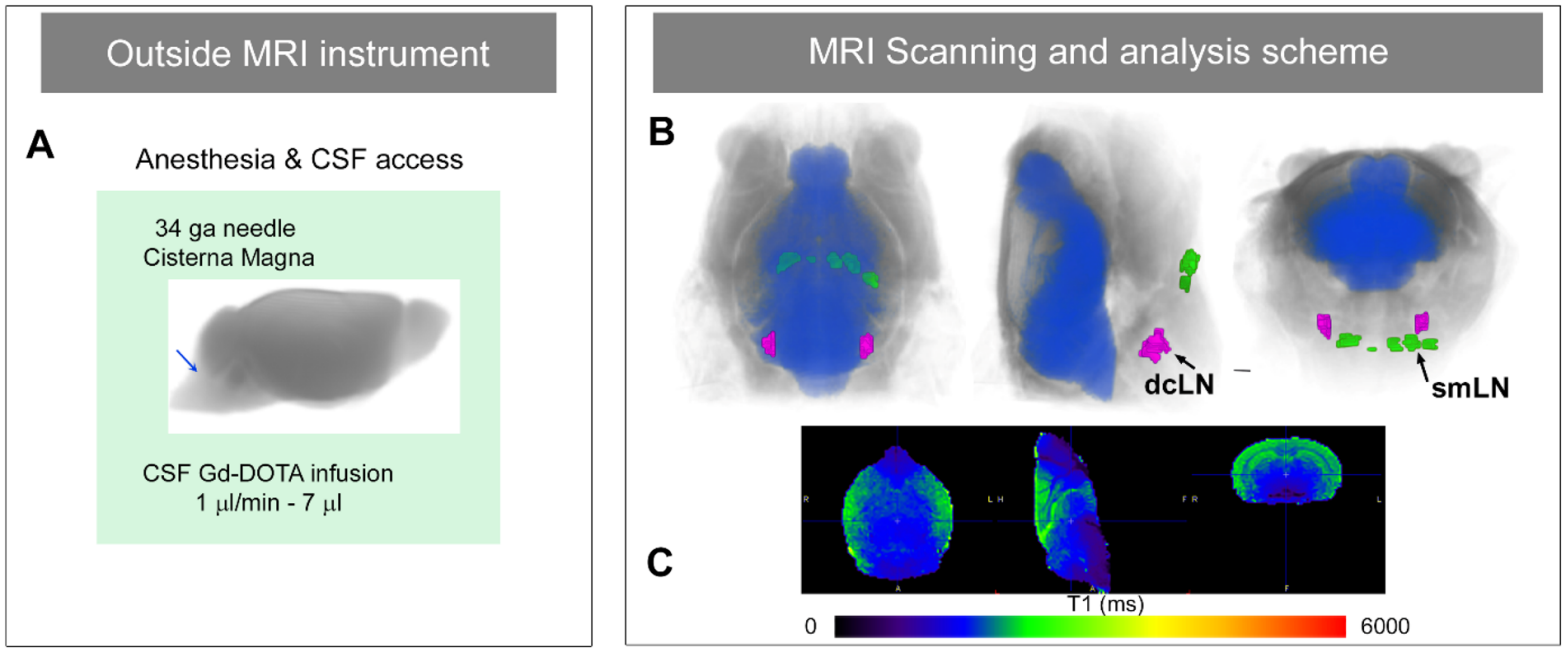
Experimental overview of glymphatic transport and cervical lymphatic solute drainage by T1 mapping. (**A**) The anesthetized mouse first receives paramagnetic contrast into the cerebrospinal fluid (CSF) via the cisterna magna on the bench outside the magnet. (**B**) The first acquisition ‘snapshot’ is obtained after ~ 1 h from the time of CSF infusion. 3D volume rendered anatomical MRI in three views are shown of a mouse head and neck highlighting the principle of capturing GS transport of Gd-DOTA into the brain (blue color) as well as drainage to the submandibular cervical lymph nodes (smLN, green color) and deep cervical lymph nodes (dcLN, magenta color) in the same image session (figure created using Amira version 6.5.0, Thermo Fischer Scientific). (**C**) Processed color-coded T1 map from the brain in three orthogonal planes of a mouse after CSF Gd-DOTA administration illustrating that GS transport of Gd-DOTA is captured as T1 reductions in the brain (figure created using PMOD software Version 3.908, PMOD Technologies LLC). Several T1 maps can be acquired post-injection and the anesthetized animal can also be allowed to recover from this procedure for longitudinal studies.

The experimental animal procedures for measuring GS transport by T1 mapping are outlined in Fig. 1. The procedures include anesthesia of the mouse and CSF administration of Gd-DOTA via the CM performed on the bench outside the MRI instrument (Fig. 1A). These procedures are followed by quantitative 3D MRI acquisitions for T1 mapping of the whole head and neck of the mouse at a prefixed time after CSF administration of tracer (Fig. 1B, C). Note that the field of view of the whole head includes both the brain and cervical lymph nodes in the neck area as shown in Fig. 1B.

### Visualization of GS transport by T1 mapping in KX anesthetized mice

**Figure 2:**
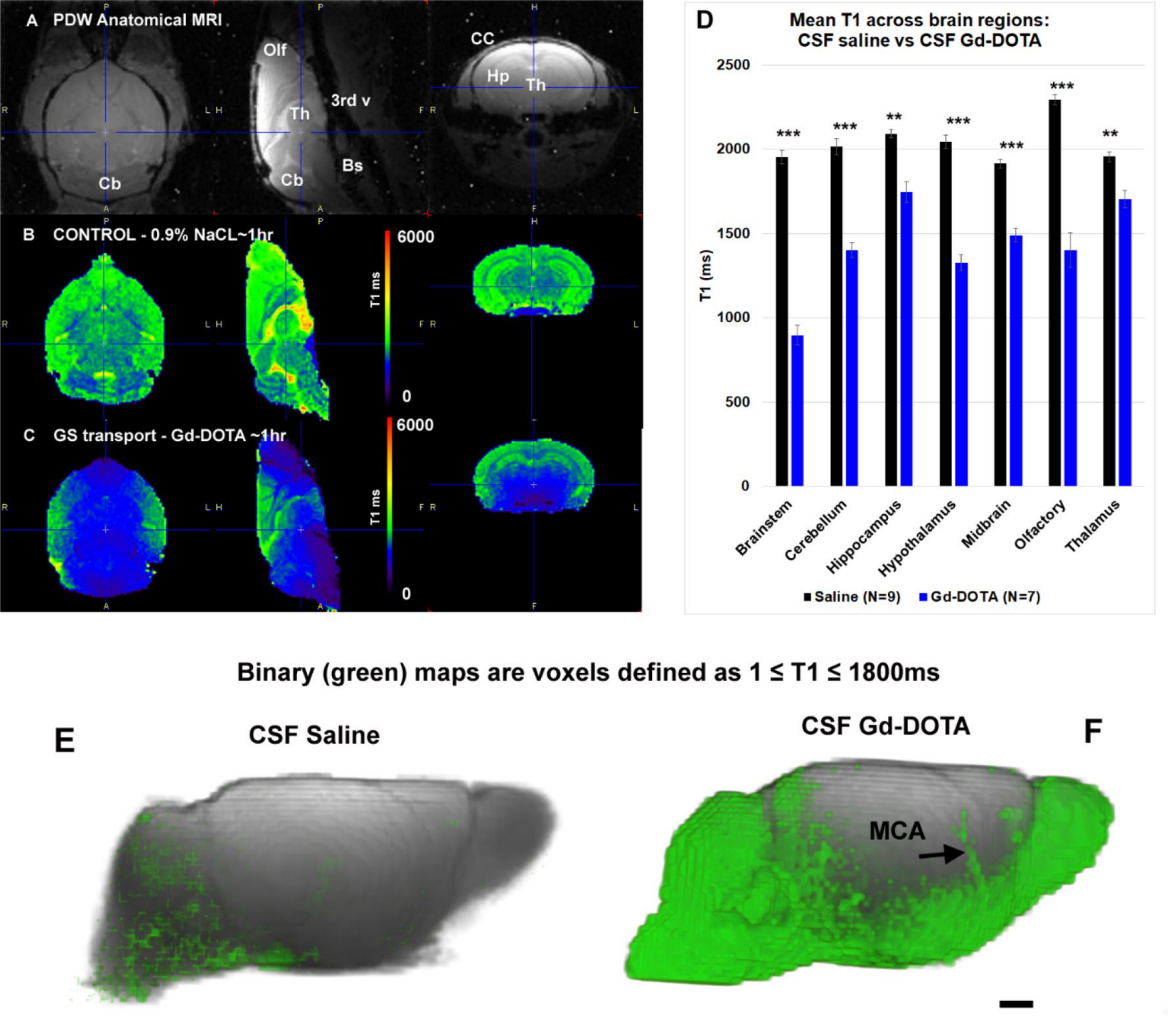
Glymphatic transport of Gd-DOTA in whole mouse brain by T1 mapping. (**A)** Proton-density weighted (PDW) MRIs of the brain from a control mouse displayed in three orthogonal planes to highlight landmarks of interest. Cb = Cerebellum; Th = Thalamus; Olf = Olfactory Bulb; 3rd V = 3rd cerebral ventricle; Bs = brainstem; Hp = hippocampus; CC = corpus callosum. Scale bar = 2 mm. (**B)** Corresponding color-coded T1 map of control mouse brain illustrating that CSF areas are characterized by high T1 values (> 3000 ms) and brain tissue by T1 values of ~ 2000 ms. (**C)** A color coded T1 map from mouse brain 1 h after Gd-DOTA into CSF. Glymphatic transport of Gd-DOTA is evidenced by the lower T1 values (blue color) in several brain regions when compared to the control mouse in B. (**D)** Statistical analysis using Welch’s t-test (unequal variance t-test), (Bonferroni correction not applied) of brain regional T1 values with the two conditions (CSF Gd-DOTA versus CSF saline) across groups revealed significant differences in all preselected regions (brainstem ***p < 0.0001; cerebellum ***p < 0.0001; hippocampus **p < 0.001; hypothalamus ***p < 0.0001; midbrain ***p < 0.0001; Olfactory bulb ***p < 0.0001; thalamus **p < 0.002). Data are presented as mean ± SEM. (**E)** Voxels in the brain with a T1 of 1–1800 ms are captured and converted into a 3D volume rendered binary map (green) in a mouse receiving CSF saline. (**F**) 3D volume rendered GS-clusters with a similar T1 threshold of 1–1800 ms converted into a binary map (green) from a KX anesthetized mouse 1 h after Gd-DOTA. In the Gd-DOTA mouse the GS-cluster of Gd-DOTA incorporates large parts of the whole brain and is also seen along the middle cerebral artery (MCA) when compared to the control mouse. Scale bar = 1 mm. Figure 2B,C were created using PMOD software Version 3.908, PMOD Technologies LLC. Figure 2E,F were created using Amira version 6.5.0, Thermo Fischer Scientific.

In the presence of paramagnetic Gd chelate contrast agents, the T1 relaxation time in the tissue is reduced in a Gd concentration dependent manner and GS transport of Gd-DOTA using T1 mapping is therefore visualized as brain areas with reduced T1 values in comparison to control mice receiving CSF 0.9% NaCl (a.k.a. ‘control’). There were no statistically significant differences between the average T1 values from the brain of the control mice anesthetized with KX or ISO (KX control (n = 5), T1 = 1994 ± 73 ms versus ISO control (n = 4), T1 = 2094 ± 88 ms, p = 0.393, Welch’s t-test (unequal variance t-test)) and all mice receiving CSF saline were therefore combined into one control group (N = 9). Figure 2 shows anatomical proton-density weighted MRIs (Fig. 2A) and the corresponding color coded T1 brain maps from a control mouse acquired 1 h after CSF saline administration (Fig. 2B) in comparison to a KX-anesthetized mouse 1 h after CSF infusion of Gd-DOTA (Fig. 2C). Anatomical landmarks are highlighted on the anatomical MRI template. In the control mouse the T1 map displays CSF filled cerebral ventricles as areas with high T1 values (T1 ~ 3000 ms) whereas normal brain tissue is characterized by T1 values in the ~ 2000 ms range (Fig. 2B). Note that white matter (e.g. white matter in the cerebellum and the corpus callosum) is distinguishable from grey matter regions because the T1 is lower in white matter.

As shown in Fig. 2C, GS transport of Gd-DOTA is clearly visible on the T1 map as areas with reduced T1 values (blue and purple colors) when compared to the control mouse (Fig. 2B). Consistent with the previously reported GS solute transport routes in rodent brain^2–4^ reduced T1 values were observed in the brainstem, cerebellum, midbrain, hypothalamus, thalamus and olfactory bulb (Fig. 2C). A one-way repeated measures ANOVA analysis with brain regions as within-subject measure in the KX + Gd-DOTA mice (N = 7) showed a significant region effect (F = 29.45; df 6, p < 0.0001) indicating that Gd-DOTA GS transport varied among regions. Gd-DOTA induced T1 decreases were largest in the brainstem (~ 50%), olfactory bulb (~ 40%), hypothalamus (~ 35%) and cerebellum (~ 27%) indicating that GS transport was highest in these areas. Figure 2D shows that the mean T1 was significantly reduced in all preselected brain regions in the KX + Gd-DOTA mice (N = 7) compared to the saline controls (N = 9) Welch’s t-test (unequal variance t-test), Bonferroni correction not applied. We also calculated the Gd-DOTA concentration in each of the preselected brain regions of the KX anesthetized mice receiving CSF Gd-DOTA based on pre-contrast T1 data derived from control mice in corresponding brain regions and post-contrast T1 from the KX + Gd-DOTA mice (for further details see Lee et al., 2018^3^). The relationship between the mean T1 and calculated concentration of Gd in each region is shown in Supplementary Fig. 1. As expected, the more GS transport of Gd-DOTA the larger the T1 reduction (Supplementary Fig. 1).

The T1 values for each preselected brain region from KX mice receiving Gd-DOTA in comparison to control mice presented in Fig. 2D represent the mean T1 of the entire brain region of interest. Since Gd-DOTA distribute heterogeneously across the brain, some of the regional T1 values include tissue voxels with normal T1 values. To improve visualizing GS transport of Gd-DOTA in the whole mouse brain on the T1 maps we selected a T1 cutoff value of 1800 ms derived from brain-wide T1 values measured in control mice receiving CSF saline and created binary maps of voxels in the range of 1 ms ≤ T1 ≤ 1800 ms. Thus, in mice receiving CSF Gd-DOTA these binary maps represent GS Gd-DOTA transport. The volume rendered 3D binary maps of voxels in the range of 1 ms ≤ T1 ≤ 1800 ms (a.k.a. ‘GS-cluster’) are shown in a control mouse 1 h after saline (Fig. 2E) compared to another mouse 1 h after Gd-DOTA (Fig. 2F). In the control mouse, the GS-cluster only captures a few voxels which are scattered in the brainstem, cerebellum, pituitary and also occasionally overlap with large vasculature as blood appears darker on anatomical SPGR images (Fig. 2E). In the Gd-DOTA mouse, the GS-cluster is evident in large parts of the brain proper (Fig. F) in a typical GS transport pattern as previously described including along the middle cerebral artery^2–4^. GS-cluster volumes in KX anesthetized mice ~ 1 h after CSF Gd-DOTA were significantly increased when compared to control mice (GS-cluster Gd-DOTA (N = 7) 51,938 ± 8,610 voxels vs GS-cluster saline (N = 9) 8,963 ± 2,522, p-value < 0.0001, Welch’s unequal variances t-test). To assess the relative variability of GS transport across individual mice, we also calculated the coefficient of variation (COV) of the GS-cluster across mice receiving CSF Gd-DOTA which was ~ 10% across the group.

### Quantification of Gd-DOTA drainage from brain/CSF to cervical lymph nodes

**Figure 3:**
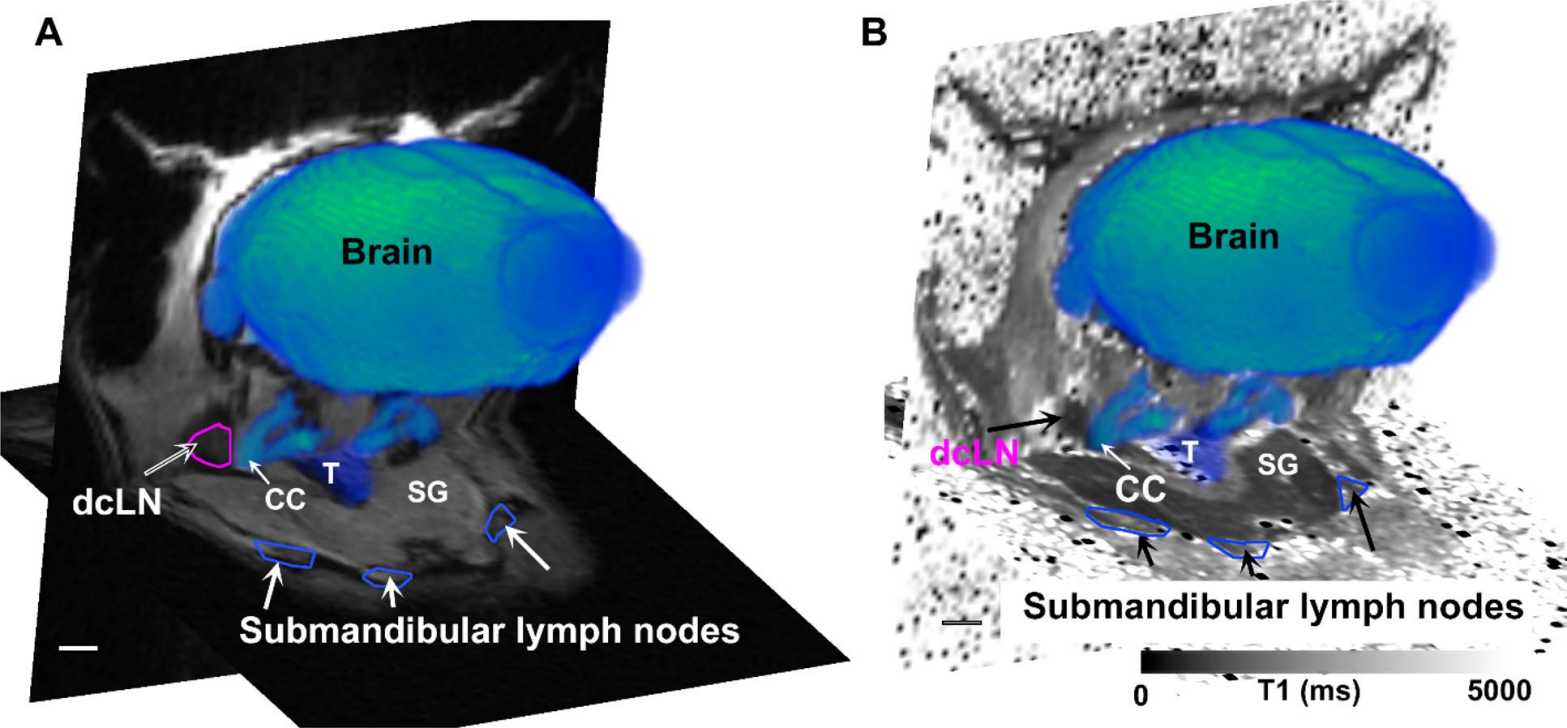
(**A**) 2D Anatomical SPGR MRIs from a KX anesthetized mouse ~ 1 h after CSF Gd-DOTA are shown at the level of the deep cervical lymph nodes (dcLN) and the submandibular lymph nodes. The brain and carotid arteries have been separately segmented, 3D volume rendered and colored in blue to illustrate their locations in relation to the lymph nodes. The dcLN are positioned dorsolateral to the trachea (T) and lateral to the common carotid (CC) just below the bifurcation. The submandibular lymph nodes are outlined in blue. (**B**) corresponding T1 map of the mouse shown in A, with the same landmarks for location of the cervical lymph nodes. Note that the deep and superficial cervical lymph nodes appear as dark rounded structure due to uptake of Gd-DOTA from the CSF/brain. SG = salivary gland; T = trachea. Scale bars = 1 mm. Figures were created using Amira version 6.5.0, Thermo Fischer Scientific.

We next explored solute drainage routes from the brain via the GS to the lymphatic system outside the skull. The deep cervical lymph nodes and the more superficially positioned smLN are reported to be major drainage sites for solute and metabolic waste from the GS system^18–20^. The spoiled gradient echo (SPGR) MRI images with low flip angles (for more details see methods) had superior signal-to-noise and contrast-to-noise ratios for anatomically localizing the submandibular cervical lymph nodes (smLN) and the deep cervical lymph nodes (dcLN) in the neck area of the C57BL6/J mice. The smLN are positioned rostro-medial to the salivary glands^21^ and are relatively large (~ 1.0–2.0 mm across) which facilitated their identification on the MRI images acquired with a voxel resolution of 0.18 × 0.18 × 0.18 mm^3^. The deep cervical lymph nodes located dorsolateral to the trachea and lateral to the common carotid artery are considerable smaller (~ 0.2–0.5 mm) (Supplementary Fig. 2). Due to their deep anatomical location and smaller size the dcLN were more difficult to localize on the MRI images in comparison to the larger and superficially positioned smLN. Figure 3A shows the locations of the dcLN and the smLN on the anatomical MRI and the corresponding T1 maps (Fig. 3B) from a KX anesthetized mouse receiving CSF Gd-DOTA. We highlighted key anatomical landmarks including the common carotid (CC) arteries, the bifurcation of the CC into the external and internal carotids, trachea (T) as well as the salivary glands (SG). Note that the dcLN are located just lateral to the CC at level of the bifurcation. The inherent signal intensity of the area associated with the lymph nodes on the SPGR MRI images is only slightly darker when compared to the surrounding muscles and fascia. The T1 maps were deliberately chosen from a mouse receiving CSF Gd-DOTA because the lymph nodes ‘darken’ after uptake of Gd-DOTA facilitating their visualization (for more information see section below). In Fig. 3 we also show the location of the submandibular nodes.

**Figure 4:**
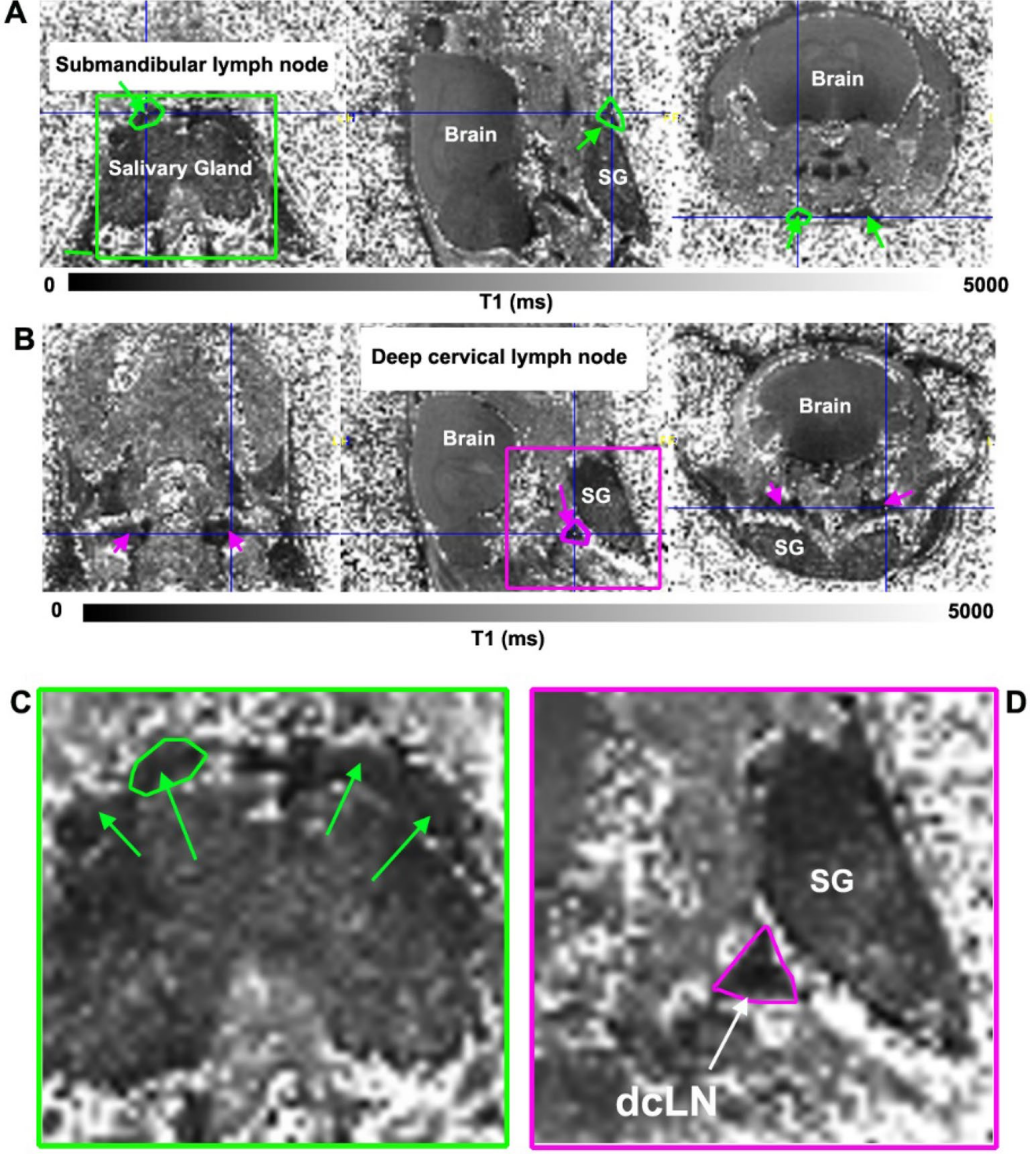
(**A**) T1 map in three orthogonal planes at the level of the submandibular lymph nodes (smLN) from a KX-anesthetized mouse ~ 1 h after receiving CSF Gd-DOTA. The smLN appear as dark rounded structures on top of the salivary gland (SG), and have darkened due to uptake of Gd-DOTA (compare with MRI data from control mice, Supplementary Fig. 3). One of the smLN is outlined in green and is clearly defined in all three anatomical planes. (**B**) T1 map from the same mouse at the level of the deep cervical lymph nodes (dcLN) which is outlined in magenta in the sagittal plane. Note that the outline of the small dcLN is best defined in the sagittal plane, but is more diffuse in the other dimensions. (**C**) Green box insert in A, magnified to better display the darkened smLN (green arrows). (**D**) Magenta box insert in B magnified to highlight the dcLN in the sagittal plane. Scale bars = 1 mm.

Figure 4 shows T1 maps in three orthogonal planes of the head and neck region at the level of the smLN (Fig. 4A) and dcLN (Fig. 4B) from a KX anesthetized mouse 1 h after receiving CSF Gd-DOTA. The smLN outlined in green are best visualized in the horizontal plane and the dcLN outlined in magenta is best visualized in the sagittal plane because of their triangular shape (Fig. 4B,D). It should be noted that the triangular structure designated ‘dcLN’ in Fig. 4B likely include afferent and efferent lymphatics and surrounding fascia covering the node. Similarly, the smLN visualized on the T1 map includes the fascia and associated lymphatics. In control mice receiving CSF saline the lymph node identification on the T1 maps were based on the anatomical MRIs and are shown in Supplementary Fig. 3. In control mice receiving saline the center of the smLN sometimes appeared brighter (Supplementary Fig. 3). In one of the KX Gd-DOTA mouse the dcLN could not be clearly identified and was not included in the analysis. Quantitative analysis showed a ~ 40% decrease in the mean T1 of dcLN in KX + Gd-DOTA mice in comparison to controls (dcLN, Gd-DOTA (N = 6) T1 = 1,093 ± 127 ms versus dcLN, saline (N = 9) = 1895 ± 281 ms, p < 0.0001, Welch’s unequal variances t-test). For the smLN there was a ~ 25% reduction with Gd-DOTA in comparison to controls (smLN, Gd-DOTA (N = 7) T1 = 1853 ± 188 ms versus smLN, saline (N-9), T1 = 2,172 ± 395 ms, p = 0.0059).

We also explored Gd-DOTA uptake patterns in individual smLN excised 1 h after CSF administration at higher spatial resolution using a 3D FLASH T1-weigthed sequence where the presence of Gd-DOTA would be evidenced as high intensity signal within the isolated smLN. Four different smLNs excised from a mouse receiving CSF saline is shown in Supplementary Fig. 4A and four different smLNs from a mouse receiving CSF Gd-DOTA is shown in Supplementary Fig. 4B imbedded and imaged in 1% agar. There is high signal intensity observed in small focal areas in the periphery of 2 of the 4 smLNs from the mouse receiving Gd-DOTA (but not in the smLN from the mouse receiving CSF saline) signifying the presence of Gd-DOTA and therefore drainage from CSF/brain and into the nodes. These results strongly indicated that Gd-DOTA drained from the GS to the smLN as has been shown for other tracer molecules using other techniques^5^. In the high spatial resolution ex situ MRI scans small*,* punctuate dark/low signal intensity areas in the smLN are also evident which likely represent small blood-filled vessels which due to the susceptibility effect from blood ‘bloom’ and appear larger in size on the MRI images. Post-mortem histology of a smLN is shown in Supplementary Fig. 4D displays lymphoid follicles in the cortex and medullary cords in the center of the node. The Hematoxylin/Eosin stained lymph nodes in Supplementary Fig. 4D also confirm the presence of the blood-filled vessels in the nodes. We were not able to obtain sufficient signal-to-noise for ex situ MRI images of the dcLN and higher resolution data of Gd-DOTA uptake are not reported in these smaller lymph nodes.

### Comparison of GS transport in KX versus ISO anesthetized mice

**Figure 5:**
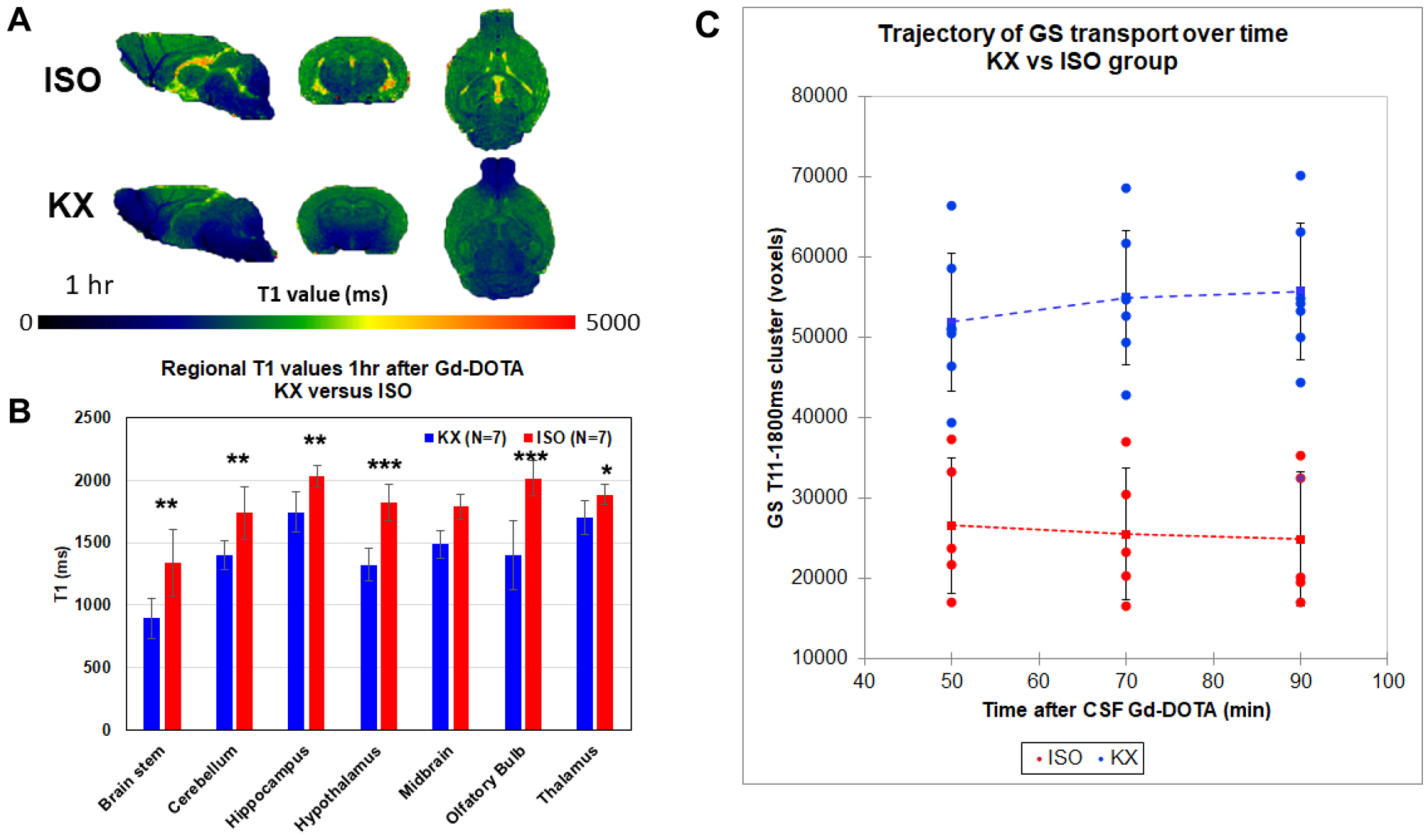
GS transport in mice anesthetized with KX is increased compared to ISO. (**A)** Color coded T1 brain maps presented in three orthogonal planes from an ISO anesthetized mouse (top) and a KX anesthetized mouse (bottom) acquired ~ 1 h after Gd-DOTA CSF administration. It is evident that there is overall less brain tissue with Gd-DOTA induced reduced T1 values in the ISO anesthetized mouse compared to the KX mouse indicating decreased GS transport with ISO. Figure 5A was created using PMOD software Version 3.908, PMOD Technologies LLC. (**B**) Welch’s unequal variances t-test was used to test T1 difference across brain regions between the two groups (KX versus ISO). Significant T1 decreases was observed in all preselected regions in the KX compared to the ISO group (brainstem **p < 0.01; cerebellum **p < 0.01; hippocampus **p < 0.01; hypothalamus ***p < 0.001; midbrain ***p < 0.001; olfactory bulb ***p < 0.001; thalamus *p < 0.05). (**C**) The GS-T1 cluster in KX (blue) and ISO (red) anesthetized mice are shown with each filled circle representing an individual mouse. The dashed blue and red lines represent the mean ± SD for the KX and ISO groups, respectively. We also compared the time course of the GS-T1 clusters from each mouse between the two groups using a repeated measures ANOVA with time as within subject factor and KX vs ISO as the between-group factor. This analysis show that the GS-T1 cluster was different between groups (p < 0.0001) over time (p < 0.01).

Color coded T1 brain maps acquired 1 h after Gd-DOTA CSF administration in Fig. 5A demonstrates that there is overall less brain tissue with reduced T1 values in the ISO compared to the KX anesthetized mouse. Statistical analysis to assess effects of anesthesia on T1 values across brain regions revealed significant decreases in all preselected regions of KX compared to ISO mice (Fig. 5B) indicating more Gd-DOTA uptake and therefore increased GS transport in the KX compared to the ISO group. Hippocampus, hypothalamus, midbrain, and olfactory bulb were regions with most significant GS transport differences between the two anesthetics (Fig. 5B).

Differences in GS transport of Gd-DOTA between ISO and KX groups were also evident when using the GS-cluster approach. Thus, at 1 h of Gd-DOTA CSF circulation, statistical analysis revealed that the whole brain GS-cluster volume in the KX mice was larger than ISO signifying enhanced GS transport with the former anesthetic (KX GS-cluster = 51,938 ± 8,610 voxels vs ISO GS-cluster = 27,765 ± 8,306, p-value < 0.001). In both the KX and ISO mice T1 maps were acquired dynamically at 50, 70 and 90 min after Gd-DOTA administration. Figure 5C shows the time-course of GS transport of Gd-DOTA (as represented by the GS-cluster) in whole brain in the KX and ISO anesthetized mice. In both groups the GS transport pattern was observed to be constant from 50–90 min signifying that for both anesthetics Gd-DOTA GS influx continued to be greater than efflux over the experimental time window and a longer washout period post-contrast will be required to see a ‘relaxing’ trajectory. We also compared the time course of the GS-T1 clusters between the two groups using a repeated measures ANOVA with time as within subject factor and KX vs ISO as the between-group factor. This analysis showed that the GS-T1 cluster was different between groups (p < 0.0001) and that this difference was sustained over time (p < 0.01).

Due to differences in respiratory patterns and significant motion artefacts in the neck area in mice anesthetized with ISO compared to KX we did not extend the anesthetic comparison analysis to the lymph nodes.

### Voxel wise analysis of GS transport via T1 mapping between KX and ISO

**Figure 6:**
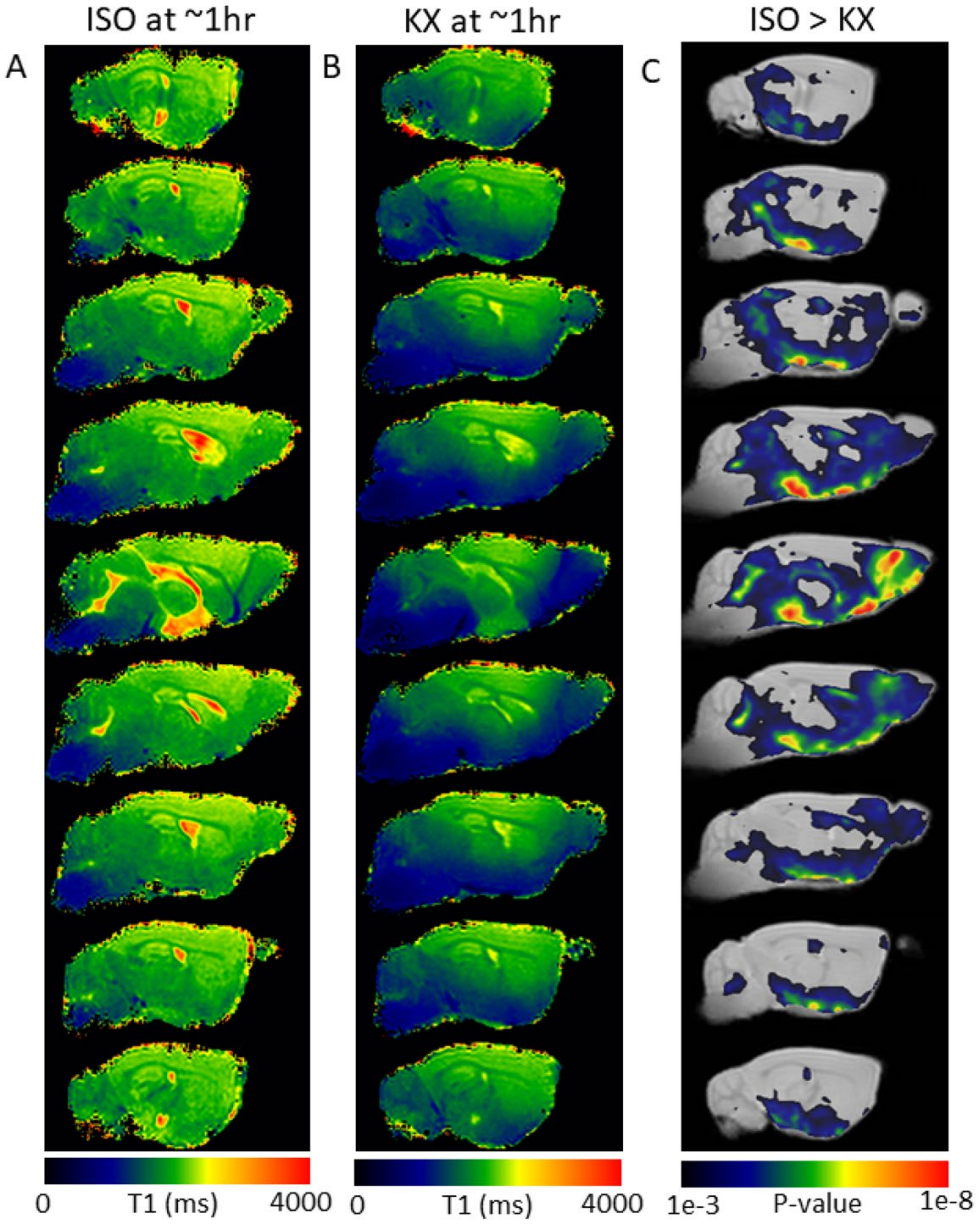
Voxel-wise analysis of GS transport differences in ISO and KX anesthetized mice. (**A)** Population averaged T1 maps of 7 different mice anesthetized with ISO 1 h after Gd-DOTA CSF administration showing GS transport in a color-coded manner. (**B)** Population averaged T1 maps of 7 different mice anesthetized with KX 1 h after CSF Gd-DOTA. Again, the color coded T1 maps show areas of high GS transport in blue colors (low T1) and no GS transport in red color (normal T1 ~ 2000 ms for tissue). (**C)** Statistical parametric maps (color coded for p-values) were calculated, corrected for FDR < 0.05 and overlaid onto population averaged proton density weighted MRI images to display anatomical areas with significantly lower values in KX compared to ISO anesthetized mice. Figures were created using SPM12 (https://www.fil.ion.ucl.ac.uk/sp) software package.

The voxel-wise analysis focused on comparing GS transport between ISO and KX anesthetized mice at 1 h after Gd-DOTA CSF administration. As shown, at 1 h after CSF Gd-DOTA the population averaged T1 maps clearly show an increased tissue area (blue color) of reduced T1 in KX anesthetized (Fig. 6B) compared to ISO anesthetized (Fig. 6A) mice. The statistical parametric maps (Fig. 6C) highlighting significantly reduced T1 voxels in KX compared to ISO anesthetized mice were largely consistent with the ROI analysis and highlight that the olfactory bulb, midbrain and hypothalamus are areas with higher GS transport of Gd-DOTA in KX mice and corroborated the results reported in the ROI and cluster analyses (Fig. 6). Note that the statistical parametric maps also depict that GS transport in the cerebellum and brainstem is largely similar between the two anesthetic regimens.

### Physiological parameters and intracranial pressure (ICP) across anesthesia groups

As expected based on previous reported data^22^ KX-anesthetized and ISO-anesthetized mice in our study exhibited different physiological profiles during MRI scanning. The mean respiratory rate of KX mice was ~ threefold higher than in ISO compared to KX anesthetized mice (KX: 176 ± 20 breath per min (bpm) versus ISO: 65 ± 6 bpm, p < 0.0001). Heart rate was significantly lower in KX mice compared to ISO mice (KX: 253 ± 22 beat per min versus ISO: 484 ± 23, p < 0.0001). Body temperature was normal and within similar range (36.5–37 °C) across groups. Intracranial pressure (ICP) performed in a separate series of mice did not reveal statistically significant differences (KX (N = 4) ICP 4.2 ± 1.5 mmHg versus ISO ICP (N = 4) 3.1 ± 1.7 mmHg, p value = 0.321) between the two anesthesia groups. Supplementary Fig. 5 shows examples from ICP measurements from a KX anesthetized mouse and an ISO anesthetized mouse recorded over 60 s illustrating that the mean ICP is similar but there are significant respiratory motion artefacts in the ISO anesthetized mouse.

## Discussion

The major result of this study shows that GS transport of Gd-DOTA can be directly quantified across brain regions in live mice using T1 mapping. Changes in T1 values across brain regions are representative of GS Gd-DOTA transport. Further, by extending the quantitative analysis to the neck region, T1 mapping also allowed for quantitative tracking of Gd-DOTA drainage from the brain-CSF compartments to the deep and superficial cervical lymph nodes in KX-anesthetized mice. We used a multiple gradient echo VFA-SPGR sequence for T1 mapping and T1 values in the brain of control mice ranged from ~ 1,800–2200 ms across regions in agreement with previously reported values acquired at 9.4 T in mice^23,24^ and rat brain^3,25^. As expected, in KX-anesthetized mice, GS transport of Gd-DOTA from the CSF compartment into brain parenchyma decreased the T1 when compared to brain parenchymal T1 values from mice receiving CSF saline. The Gd-DOTA induced T1 decreases were most significant in brainstem, cerebellum, midbrain and olfactory bulb which is the regional pattern typically observed for GS transport in rodent brain^2–4^.

In GS transport studies using DCE-MRI, a typical experimental protocol entails MRI scanning before, during and several hours after CSF Gd-contrast administration^2–4^. This is necessary because pre-contrast T1-weighted MRIs are needed to calculate contrast enhancement ratios for post-hoc quantitation of GS transport kinetics^2,3,12^. Long-lasting MRI studies in anesthetized mice with indwelling CSF catheters can be technically challenging and GS transport studies in mice are most often shorter (~ 1 h) than in those conducted in larger rats and GS transport kinetic parameters not reported^5,26^. In the experiments conducted here, the CSF catheter was removed from the cisterna magna and the skin closed simplifying the set-up for MRI scanning. The T1 mapping technique introduced here circumvented the need for pre-contrast baseline scans and by implementing a pre-fixed time interval from CSF contrast administration to start of the MRI acquisition allowed for comparisons of GS transport within and across experimental groups. Of note, Watts et al.^27^, were the first to use 3D VFA-SPGR T1 mapping similar to the sequence used here to quantify GS solute transport by T1 mapping in human brain. Specifically, after intrathecal lumbar injection of gadobutrol, they reported that gadobutrol uptake in the human brain was notable (via T1 tissue reductions) at 4 h after contrast injection and peaked between 10 and 26 h reaching concentrations at ~ 0.1 mmol/L^27^. Watts et al. did not capture drainage to cervical lymph nodes by T1 mapping but documented complete clearance of MR contrast from the entire brain ~ 3 days later^27^. In other human studies using DCE-MRI GS transport parameters such as influx and efflux are carried out by normalizing the post-contrast T1-weighted brain images to a reference region such as the posterior part of the superior sagittal sinus^10,28^. This semi-quantitative approach has also enabled detection of solute (gadobutrol) from the human brain to the cervical lymph nodes^29^. However, to the best of our knowledge the data presented here are the first to extend T1 mapping of brain-wide GS transport to also capture deep and superficial cervical lymph node drainage concurrently in the same animal. The ability to measure GS transport and cervical lymph node drainage in the same animal longitudinally is advantageous and time efficient as the interplay between the glymphatic and lymphatic system can be studied in healthy brain as well as in disease stages and translated to human studies as well.

To test the sensitivity of T1 mapping for tracking changes in GS transport efficiency we compared GS transport patterns in mice anesthetized with KX versus ISO anesthesia. Based on previous reports^22^, one would expect reduced Gd-DOTA uptake in ISO-anesthetized mice compared to KX-anesthetized mice and therefore less T1 tissue reductions with the former anesthetic and our data confirmed this previous observation. The dynamic studies of KX- and ISO anesthetized mice were presented using the binary GS-T1 clusters which captures GS transport of Gd-DOTA based on T1 values in the range of 1–1800 ms. Using this approach, we did not observe a ‘peak’ and clearance of Gd-DOTA which are typically observed in rodent studies using the DCE-MRI and CSF contrast administration^2–4,17^. However, there are alternative ways of displaying the T1 data observed and mean T1 values from some of the brain regions (e.g. olfactory bulb) exhibited ‘peak’ and a clearance trend (data not shown). More in-depth kinetic analysis with varying Gd-DOTA ‘mass’ administration into the CSF will help further improve the T1 mapping approach presented here.

We also introduced a voxel-based atlas analysis approach to compare brain-wide GS transport across the two different anesthesia group which provided a statistical approach to pin-point differences across the whole brain at the voxel level. The statistically significant areas revealed in ISO > KX show that the olfactory bulb, hypothalamus and midbrain are regions with most accelerated transport in KX compared to ISO mice. The underlying mechanisms for decreased GS transport in mice anesthetized with ISO compared to KX has been ascribed to differences in high EEG delta power and heart rate^22^. Specifically, lower heart rate and less EEG delta power associated with ISO anesthesia interferes negatively with CSF transport into the brain when compared to KX anesthesia^22^. The KX anesthetized mice in our study were also characterized by lower HR compared to ISO which might explain the GS transport differences between the two anesthetics. Paradoxically, other GS studies have shown that high cardiac pulsatility increases GS transport^30,31^ suggesting ISO anesthesia would be more efficient for GS transport compared to KX. Clearly, other complex vascular and hemodynamic parameters including vasomotion^32–34^ varies across anesthetic regimens and might be of more importance for regulating GS transport and should be explored further in future studies. Of note, the respiratory rate was significantly higher in KX compared to ISO anesthetized mice. Lower than normal respiratory rate in ISO might be associated with mild hypercarbia as has been reported for other anesthetics in spontaneously breathing rats^17^ and could contribute to decreased GS transport observed with ISO compared to KX as uncorrected hypercarbia would cause higher than normal intracranial pressure (ICP). We did not measure arterial or end-tidal pCO_2_ in the two groups of mice anesthetized with KX and ISO. However, instead ICP was measured in parallel groups of mice under the same physiological conditions and observed no statistical differences in ICP between the two anesthetics. We therefore concluded that potential mild hypercarbia (and increased ICP) was unlikely to explain the observed differences in GS transport.

The other major finding of our study revealed that drainage of Gd-DOTA to the dcLN and smLN was detectable in vivo by T1 mapping in KX anesthetized mice. Our data are in agreement with Ma et al., who used optical imaging techniques and described rapid drainage of the pegylated fluorescently tagged tracer P40D680 (MW 40 kDa) to the deep cervical and submandibular lymph nodes of the neck of mice^20^. Specifically, Ma and colleagues used optical techniques and demonstrated that 30-min after CSF tracer administration the signal was detectable in the smLN^20^. We acquired the T1 map ~ 1 h after CSF administration of Gd-DOTA and were able to quantify a significant T1 decrease in both smLN and dcLN when compared to control mice receiving CSF saline. In comparison to control mice receiving CSF saline the T1 in the dcLN were reduced by 40% and the smLN were reduced by 25% after Gd-DOTA. These data suggest that the dcLN drain more Gd-DOTA in comparison to the smLN as have been shown for other tracer molecules using optical imaging techniques^5^. However, more investigation on the kinetics of Gd-DOTA pertaining to the lymph nodes are needed to fully establish the draining patterns between the superficial and deep lymph nodes. To further explore the contrast drainage, we dissected the smLN in live KX-anesthetized mice and performed ex vivo scanning. These scans confirmed Gd-DOTA uptake in small zones in the periphery of the smLN likely corresponding to the subcapsular areas in conjunction to afferent lymph vessels previously documented using other imaging and histological techniques^5,18,35,36^.

## Limitations

Due to significantly more respiratory related motion artefacts in the neck region including smLN and dcLN in mice receiving Gd-DOTA and anesthetized with ISO we did not extend the T1 mapping analysis to explore differences in Gd-DOTA drainage between the two anesthesia groups. The spatial MRI image resolution of ~ 180 micron^3^, inevitable respiratory motion in the neck areas of interest and partial volume averaging effects interfered negatively with cervical lymph node visualization especially in the ISO anesthetized mice. Thus, in ISO anesthetized mice motion artefacts caused by respiration adversely influenced the data quality. Respiratory artefacts were most pronounced in the ISO anesthetized mice because inhalational anesthetics cause paradoxical respiratory motion when compared to KX anesthesia. We did not implement mechanical ventilation in the study design because positive pressure ventilation influences CSF flow patterns and therefore overall GS transport. Respiratory gating was also not implemented as this would have caused different scan times for the two groups due to the significant differences in respiratory rate. Specially, the respiration of the anesthetized mouse trigger controls the MRI image acquisition thereby avoiding data collection during the period of diaphragm movement (i.e. inspiration phase). However, as reported in this study, KX anesthetized mice have nearly twice as high respiration rate (~ 120 bpm) compared to ISO (~ 60 bpm) therefore in the setting of respiratory gating, the KX animals would have twice as many non-data collection periods compared to ISO anesthetized mice. Consequently, the total scanning time for KX anesthetized mice would be substantially longer than for ISO and we would not be able to capture T1 map over the same time frame for both anesthetics which would confound our data comparison between groups*.* In addition, we positioned the rf surface coil under the head (and therefore far away from the smLN in the superficial neck area) of the mouse to optimize the capture of brain-wide GS transport. Alternate rf coil designs and coil position closer to the neck area will undoubtedly improve overall signal to noise in the MRI images in future studies and should be explored for better visualization of cervical lymph nodes.

## Conclusions

In summary, the in vivo T1 mapping approach allows for quantification of GS transport in the whole mouse brain at the voxel level without the need for pre-contrast scans. Comparisons of GS transport of the paramagnetic contrast solute Gd-DOTA across mice within and across experimental groups was accomplished by timing the onset of MRI scanning in relation to the time of CSF Gd-DOTA administration. In addition, drainage of Gd-DOTA from the brain to the deep cervical lymph nodes and submandibular lymph nodes was also captured in KX anesthetized mice breathing spontaneously by quantifying T1 changes in comparison to the control mice receiving CSF saline. The ability to measure GS transport and cervical lymph node drainage in the same animal longitudinally is advantageous and time efficient as the coupling between the glymphatic and lymphatic system can be studied and the T1 mapping technique is easily translated to human studies.

## Methods

All animal experiments were approved by the Institutional Animal Care and Use Committee at Yale University in accordance with the United States Public Health Service’s Policy on Humane Care and Use of Laboratory Animals. Twelve-week old male C576BL/6 J mice (23–30 g) mice were purchased from the Jackson Laboratory (Bar Harbor, Maine, US). The experiments were designed to first assess the T1 mapping technique for tracking GS transport of Gd-DOTA in Ketamine/Xylazine (KX) anesthetized mice in comparison to KX anesthetized control mice receiving 0.9% NaCl (KX + saline) into CSF. Isoflurane (ISO) has previously been shown to reduce GS transport in mice when compared to KX anesthetized mice^22^. Therefore, to further test and validate the T1 mapping approach for GS transport we also implemented groups of mice anesthetized with ISO (ISO + Gd − DOTA) and control mice anesthetized with ISO receiving CS saline) to test the hypothesis that T1 mapping can track decreases in solute GS transport induced by ISO in comparison to KX. Finally, in a separate series of KX and ISO anesthetized mice intracranial pressure was measured during the two different anesthetic regimens.

### Anesthesia

All mice were induced with 3–4% isoflurane. After loss of the righting reflex, KX-anesthesia mice received an intraperitoneal (IP) injection of KX mixture: (ketamine 17.5 mg/ml and xylazine 2.5 mg/ml, 0.1 ml/20 g body weight) with glycopyrrolate (0.2 mg/kg IP). KX anesthesia was maintained with KX (0.05 ml of the KX-mixture/20 g body weight) administered every 30 min via an IP catheter; and supplemented with 1:1 air:O_2_ mixture. ISO-anesthetized mice received glycopyrrolate (0.2 mg/kg IP) and anesthesia was maintained with 2–2.5% isoflurane (ISO) delivered in a 1:1 air:O_2_ mixture. All mice were allowed to breathe spontaneously. Physiological parameters including respiratory rate, body temperature, and heart rate were continuously monitored using MRI compatible monitors (SA Instruments, Inc., Stony Brook, NY). Body temperature was maintained between 36.5 ~ 37.5 °C by a heating pad.

### Infusion of Gd-DOTA or saline into the CM

The anesthetized mice were positioned in a stereotaxic frame and their neck slightly flexed with care to not compromise the airway and spontaneous breathing pattern. Through a midline incision, the atlanto-occipital membrane was exposed and a 34-ga shortened needle (Hamilton, US) connected via polyurethane tubing to a 50 µl Hamilton syringe (Hamilton, US) mounted in a micro-infusion pump (Legato 130, KD Scientific, Holliston, MA, USA) was inserted into the CM. Previous studies reporting on tracer delivery for GS transport studies in mice via the CM have used infusion rates of 0.1–2 µl/min for a total volume of 5–10 µl^22,37–40^. In this study we infused 7 µl of Gd-DOTA (Guerbet LLC, Princeton, NJ, US) prepared as a 1:20 dilution in sterile 0.9% NaCl or 7 µl of sterile 0.9% NaCl at an infusion rate of 1 µl/min. After the infusion, the needle was left in place for 2-min, after which it was removed, and the dura sealed with cyanoacrylate glue. Collectively, anesthesia, CM exposure and Gd-DOTA infusion required ~ 30 min.

### Dissection and histology of cervical submandibular lymph nodes

KX anesthetized mice received CSF Gd-DOTA (N = 3) or CSF saline (N = 3). After ~ 1 h of CSF circulation the submandibular cervical lymph nodes (smLN) were dissected and positioned in an Eppendorf tube containing 1% agar. Following MRI, the smLN were drop fixed in 10% neutral buffered formalin overnight at 4 °C in preparation for histology. The lymph nodes were then washed briefly in PBS and transferred to 70% ethanol before being paraffin embedded. Sections were cut (5 µm) using a microtome, mounted and stained with hematoxylin/eosin. 20X images were captured using the Keyence BZ-X700 microscope system. C57BL/6 mice (N = 3) were anaesthetized by ketamine/xylazine injection i.p., and then received 5 μl of 1% Evans blue (Sigma-Aldrich) into the cisterna magna. Thirty minutes after injection, anesthetized mice underwent dissection for assessment of Evans blue content at the level of the deep cervical lymph nodes (dcLN). After location of the blue-colored dcLN, a small fiduciary marker (1 mm PE tubing) was placed next to the node and the mouse underwent MRI scanning (see below) for anatomical landmarking of the dcLN and surrounding structures.

### Intracranial pressure (ICP) measurements

ICP was measured in separate series of mice anesthetized with either KX (N = 4) or ISO (N = 4). A pre-calibrated optical pressure transducer with an outer diameter (OD) of 0.42 mm (Samba Preclin420 LP, Samba Sensors AB, Sweden) was used for the ICP measurements in the CM. For ICP measurement a closed system was created consisting of the pressure sensitive optical transducer inserted into a water-filled 50μL Hamilton syringe (Hamilton, US) connected to a 34-guage needle (Hamilton, US). After anesthetization with either KX or ISO, the head of the mouse was secured in the stereotaxic frame and the 34G needle was inserted into CM as described above. Once inserted the optical probe was kept in place and the pressure was continuously measured for 5 min at 150 samples per second using a two-channel control unit (Samba control unit 202, Samba SensorsAB, Sweden). Physiological parameters including heart rate, respiratory and body temperature was recorded in the animals during the ICP measurements.

### MRI imaging

All MRI acquisitions were performed on a Bruker 9.4 T/16 MRI instrument with a BGA-9S-HP imaging gradient interfaced to a Bruker Advance III console and controlled by Paravision 6.1 software (Bruker Bio Spin, Billerica, MA, USA). **In vivo experiments**: To obtain consistent animal positioning relative to the radio frequency (RF) coils and imaging gradients, a custom 3D printed animal cradle for supine positioning was designed and fabricated using acrylonitrile butadiene styrene plastic. A volume RF transmit with an inner diameter (ID) of 5.0 cm and a 10 mm surface receive coil was utilized for all in vivo MRI scans. The 3D T1 mapping technique implemented in this study was based on our previously described method developed for rat brain^3,13^. In all mice, the first scan was pre-fixed to start 50 min from initiation of CSF infusion on the bench which would provide the first T1 map at ~ 1 h after infusion. Briefly, following an anatomical localizer along three orthogonal planes, a spatial inhomogeneity profile of the RF transmit (B1+) was acquired using a double angle method using a rapid acquisition with relaxation enhancement (RARE) sequence (TR = 10,000 ms, TE = 22 ms, Average = 1, RARE factor = 4, number of slices = 36, in plane resolution = 0.24 mm/pixel, slice-thickness = 0.3 mm, slice gap = 0.2 mm Flip angles = 70° and 140°). Subsequently, acquisition of 3D T1 mapping was performed using a spoiled gradient echo VFA-SPGR method (TR = 16 ms, TE = 3 ms, Average = 1, scanning time = 2 min 40 s, matrix = 100 × 100x100 reconstructed at 0.18 × 0.18x0.18 mm). A set of six flip angles (2°, 5°, 10°, 15°, 20°, 30°) were acquired for post-contrast T1 maps at 50, 70, and 90 min post infusion of Gd-DOTA and each scan required ~ 16 min. Ex situ MRI of cervical submandibular lymph nodes: The SmLN positioned within 1% agar in the Eppendorf tube was placed and secured in a RF volume coil. A 3D T1-weighted FLASH sequence with the following parameters: TR = 27 ms; TE = 5 ms, FA = 15°, NEX = 32; isotropic voxel resolution of 50 × 50x50 µm^3^ was used to screen the smLN for the presence of Gd-DOTA.

### MRI imaging processing and analyses

3D T1 maps were calculated using the linearization of the SPGR signals and unweighted least square fit as described previously^3^. In house software was written for all processing in Matlab 2017 (Mathworks, MA, USA) unless specified. T1 maps were filtered and T1 values > 5000 ms were excluded. Anatomical MRIs comprised the proton density weighted (PDW) image calculated from the 3D VFA-SPGR T1 M0 image as well as the summed low flip angle (2° and 5°) SPGR images referred to as ‘SPGR-low FA’. Analyses of in vivo MRI data included characterizing T1 differences across brain regions and cervical lymph nodes, whole brain T1-cluster analysis, reliability of the GS-transport technique across mice using the coefficient of variance, and comparisons between KX and ISO groups using ROIs and voxel-wise analysis. ROI masks of brain regions: The C57BL6/J in vivo atlas^41^ was used as a template and the anatomical PDW images were rigidly re-aligned and resliced to match the atlas and further manually edited to improve the accuracy of ROIs to capture the perimeters of anatomical landmarks including hippocampus, thalamus, cerebellum, midbrain, hypothalamus, olfactory bulb and brainstem. The edited ROIs were then superimposed onto T1 maps to extract post contrast T1 values. The mean T1 for each region was calculated over the 90 percentiles of all T1 values within the ROI. ROI for cervical lymph nodes in vivo: The anatomical SPGR-low FA MRI had superior signal- and contrast-to-noise ratios for soft tissue and were used for localizing the position of the dcLN and the smLN (Fig. 3). The dcLN and the SMLN were outlined manually using Amira software (Amira, version 6.4, Thermo Fisher Scientific, USA) on the anatomical MRIs by two team members with expertise in mouse anatomy who were blinded to the study groups; and these were subsequently transferred to T1 maps to extract post-contrast T1 values. The mean T1 for each region was calculated over the 90 percentiles of all T1 values within the ROI. GS transport analysis via T1 cluster analysis: In addition to the analysis of Gd-DOTA uptake across brain regions we also quantified GS transport by selecting voxels with a 1 ms ≤ T1 ≤ 1800 ms of the T1 maps and converting the total cluster (a.k.a. ‘GS T1 cluster’ into a 3D volume rendered binary map. Based on T1 values measured from control mice (receiving CSF saline) normal T1 values were in the range of ~ 2000 ms. Thus, voxels with a 1 ms ≤ T1 ≤ 1800 ms represent GS transport of Gd-DOTA over a given time frame.

### Voxel-wise image analysis

The 3D PDW image calculated from the 3D VFA-SPGR T1 calculation was analyzed to derive spatial registration parameters using the SPM12 (https://www.fil.ion.ucl.ac.uk/spm) software package. PDW image intensity inhomogeneity caused by the RF receive coil (B1-) was first corrected by the N4 algorithm^42^ and subsequently segmented into GM, WM and CSF compartments using the unified segmentation algorithm^43^. Publicly available 8–12-week-old C57BL/6 mice tissue probability maps (tpm) (https://www.bmap.ucla.edu/portfolio/atlases/Mortimer_Space_Atlas) were used for this purpose. Segmented images were then population averaged to create a study specific tpm as previously described^44^ and re-segmented the PDW images using affine only transform. Resulting spatial registration parameters in PDW were then applied onto corresponding T1 and further smoothed by isotropic 0.4 mm full width half maximum Gaussian smoothing kernel. Voxel-wise T1 analysis was performed by a t-test in the framework of general linear model.

### Statistical analysis

The data are presented as mean estimates for regional T1 values or GS-clusters with standard deviations (SD). For experiments with repeated measurements (e.g. scan time points) we used a repeated measures analysis of variance (RM-ANOVA). Pairwise comparisons between groups (for example, KX + Gd − DOTA versus KX + Saline or KX + Gd − DOTA versus ISO + Gd − DOTA) for regions of interest (ROI), GS-clusters at single scan time points were investigated using Welch’s t-test. The p-values were not adjusted for multiple comparisons. All t-tests are two-sided. In addition, the regional differences were corroborated with Statistical Parametric Mapping using the SPM12 (https://www.fil.ion.ucl.ac.uk/spm) software package. To assess the relative variability of GS transport via the GS-cluster across individual mice in the KX + Gd − DOTA mice the coefficient of variation (COV) was calculated according to the formula: COV = (SD/Mean) × 100%. Statistical analysis was carried out using XLSTAT statistical and data analysis solution. (Boston, USA. https://www.xlstat.com).

## Supplementary information

Supplementary information
