# Supplementary material for: In vivo T1 mapping for quantifying glymphatic system transport and cervical lymph node drainage
Source: Sci Rep. 2020 Sep 3;10:14592. doi: 10.1038/s41598-020-71582-x (PMC7471332; doi:10.1038/s41598-020-71582-x)
Supplement: Supplementary file 1 — Supplementary information [file 41598_2020_71582_MOESM1_ESM.pdf]

Supplementary Information for "In vivo T1 mapping for quantifying glymphatic system transport and cervical lymph node drainage"

Yuechuan Xue[1,2], Xiaodan Liu[1], Sunil Koundal[1], Stefan Constantinou[1], Feng Dai[3], Laura Santambrogio[3], Hedok Lee[1+], Helene Benveniste[1+*]

1. Department of Anesthesiology, Yale School of Medicine, New Haven CT, 06520, USA
2. Department of Critical Care Medicine, Xiangya Hospital, Central South University, Changsha, Hunan, 410008, China
3. Yale Center for Analytical Sciences, Yale School of Public Health, New Haven, CT
4. Englander Institute of Precision Medicine, Department of Radiation Oncology, Physiology and Biophysics, Weill Cornell Medicine, New York, NY, 10065

*Corresponding author
[+]Co-Senior author

Corresponding author:

Helene Benveniste, MD, PhD
Department of Anesthesiology
Yale School of Medicine
330 Cedar St, TMP 3
New Haven CT, 06520, USA
Telephone: 203 737 1516
Email: helene.benveniste@yale.edu

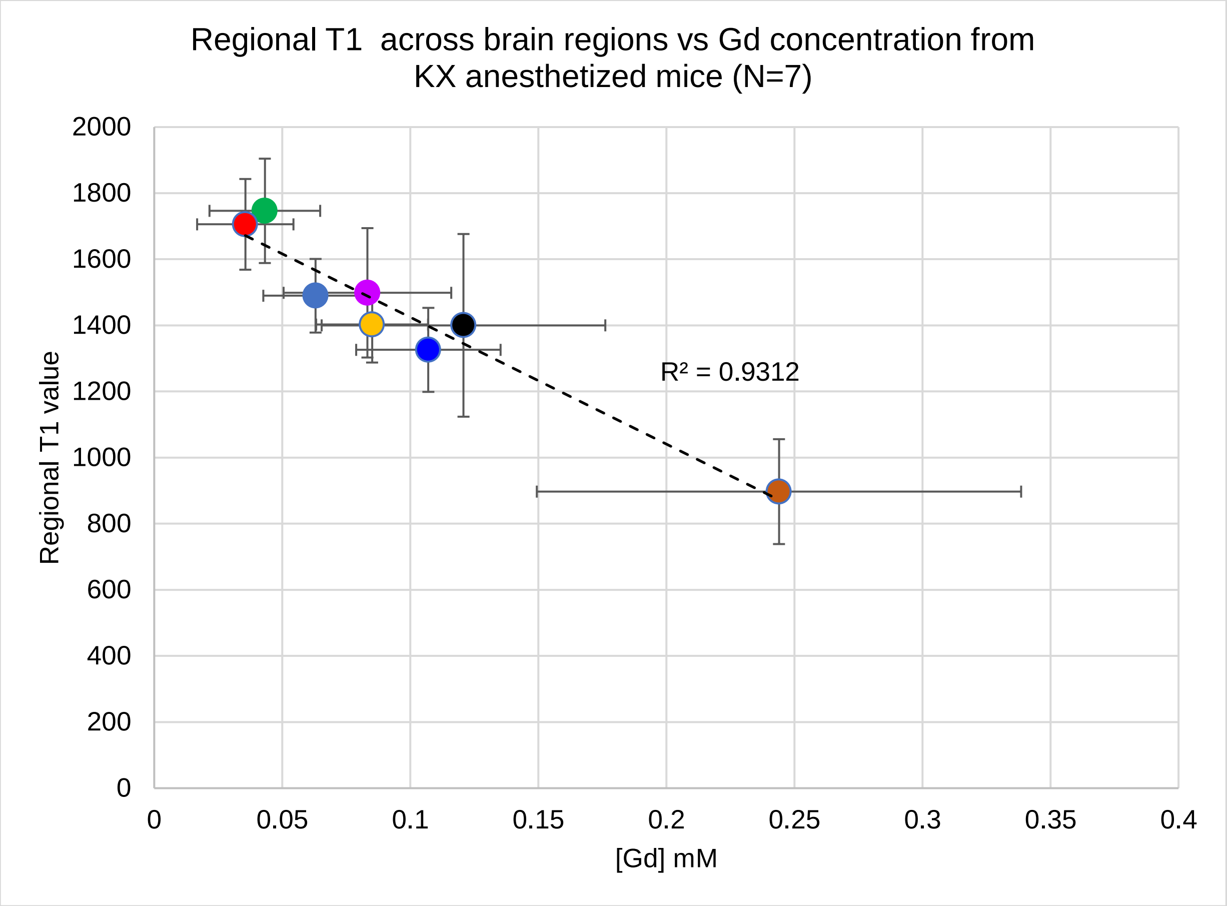

Supplementary Fig. 1: *The mean T1 from each region was extracted from eight different brain regions and converted into a correspoding Gd tissue concentration ([Gd]) based on the following equation:*

$$\frac{1}{T1_{post}} = \frac{1}{T1_{pre}} + n_{Gd}C_{Gd}$$

where $T1_{pre}$ *is the pre-contrast T1 derived from the saline control mice,* $T1_{post}$ *is the post contrast T1 ~1hr after CSF Gd-DOTA,* n_{Gd} *is the Gd-DOTA tissue concentration (mM) and* C_{Gd} *is the Gd-DOTA relativity constant for 9.4T (*$C_{Gd} = 2.69 mM^{-1}s^{-1}$*).*

Each filled circle represents a brain region: red = thalamus, green = hippocampus, light blue = midbrain, magenta = basal forebrain, yellow = cerebellum, blue = hypothalamus, black = olfactory bulb, brown = brain stem. As can be seen there is an inverse linear relationship between the T1 and the estimated [Gd] in agreement signifying that the T1 is reduced in a [Gd] dependent manner.

Data are mean $\pm$*SD.*

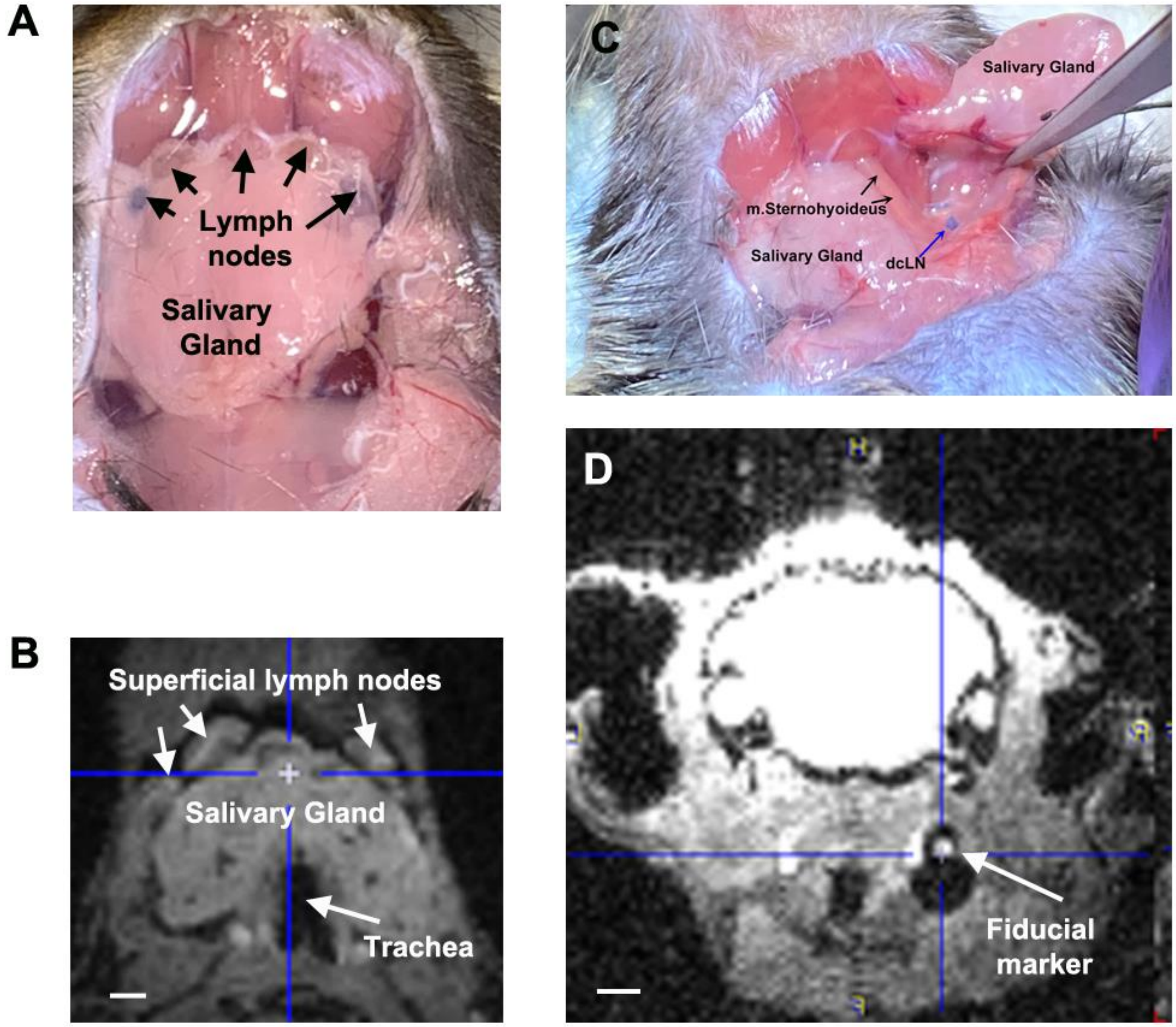

Supplementary Fig. 2: Anatomical localization of cervical lymph nodes of the mouse.
A: *Superficial (submandibular) lymph nodes and salivary gland (ventral view) of a mouse post-mortem. The skin has been removed exposing the salivary gland and lymph nodes positioned rostral to the salivary gland. Two of the 5 nodes appear blue from uptake of 1% Evans Blue administered into the CSF (30 min circulation). The nodes are ~1-1.5mm across.* ***B:*** *Anatomical MRI of an anesthetized mouse at the level of the salivary gland and superficial cervical lymph nodes.* ***C:*** *Photograph of anesthetized mouse with exposure of the left deep cervical lymph nodes (dcLN) located dorsolateral to the trachea (covered here by the sternohyoideus muscle). The left salivary gland has been moved out of the field of view to expose the dcLN. The dcLN is blue because Evans Blue has drained from the CSF/brain via the afferent lymphatics to the dcLN.* ***D****: Proton-density weighted MRI from a live, anesthetized mouse at the level of the cerebellum and neck after fiduciary marker was placed next to the left dcLN. The orthogonal blue cross-hair is at the center of the fiduciary marker. The dcLN next to the fiducial marker is not visible due the susceptibility induced signal void in the area from the phantom, which was used to mark the approximate location. Scale bars = 1mm.*

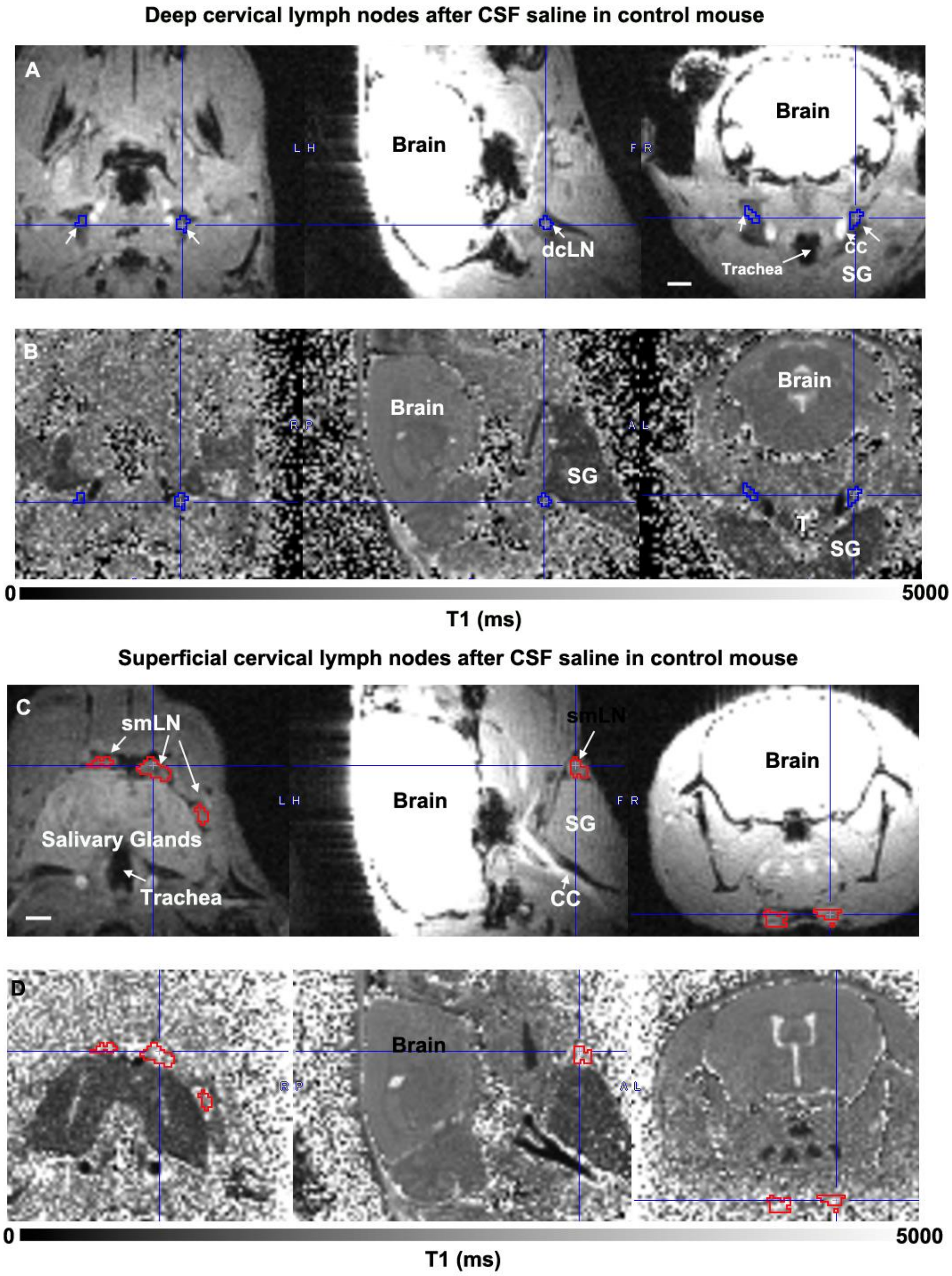

Supplementary Fig. 3: A: *Anatomical MRIs from KX anesthetized mouse ~1hr after CSF saline showing the location of the deep cervical lymph node (outlined in blue) lateral to the trachea and the common carotid (CC).* ***B****: Corresponding T1 map from the same mouse showing the outline of the dcLN as a area with a T1 value ~2000ms (similar to the T1 of the normal brain).* ***C****: Anatomical MRIs at the level of the submandibular glands (smLN) outlined in red.* ***D:*** *corresponding T1 map of the same mouse showing that the T1 value in the area associated with the smLN appear slightly brighter than that of the salivary gland and brain.*

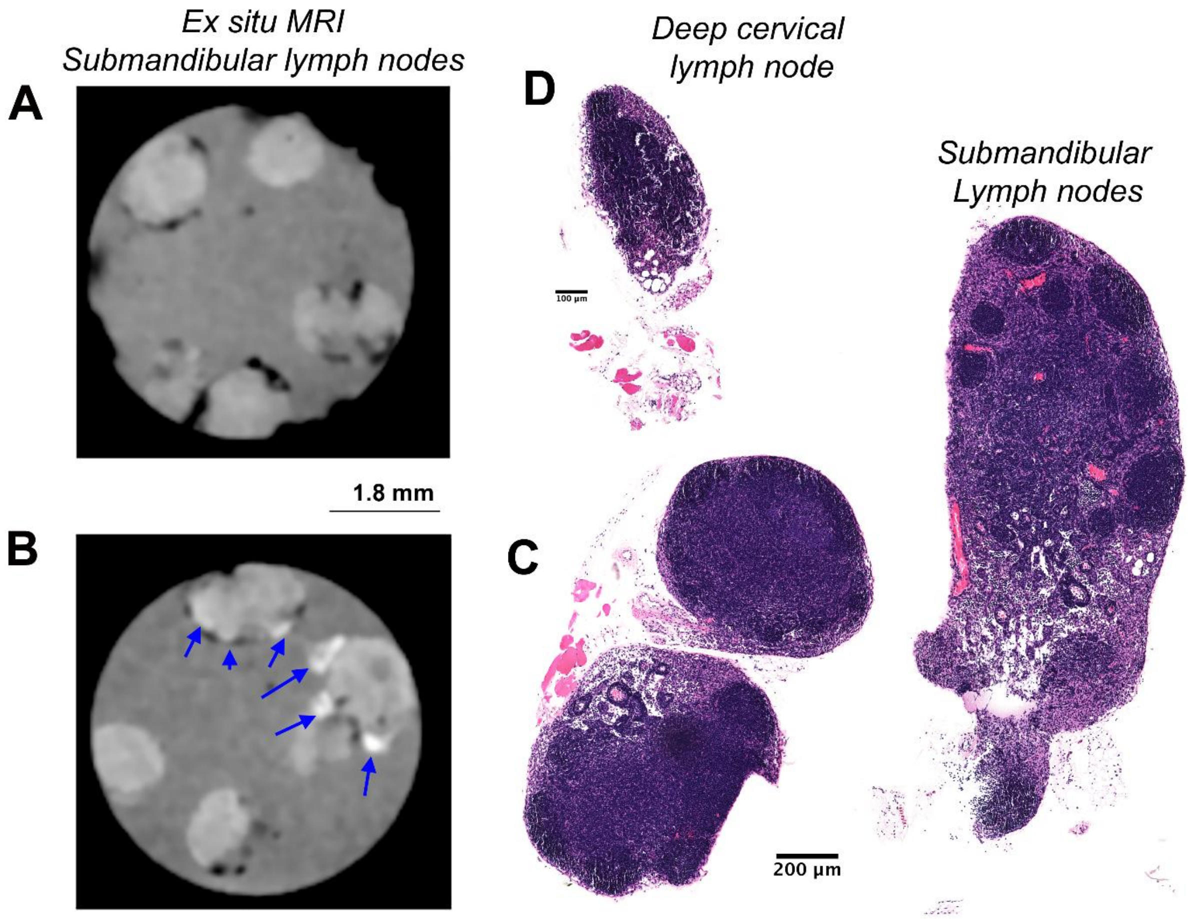

Supplementary Fig. 4: A: *2D T1-weighted MRIs of four individual SMLN dissected from a CSF saline control mouse showing that the individual lymph nodes are round-like structures characterized by a homogeneous MR signal after excision. The spotty low intensity signal areas associated with the individual nodes represent small blood vessels.* ***B:*** *Corresponding SMLN from a mouse receiving CSF Gd-DOTA. In these nodes, smaller areas with high signal intensity are observed in the periphery of 2 of the SMLN (blue arrows) signifying the presence of Gd-DOTA.* ***C:*** *Hematoxylin Eosin stain of three different submandibular lymph nodes which displays lymphoid follicles in the cortex and medullary cords.* ***D****: For size comparison we also show a H&E stained section from a resected deep cervical lymph node.*

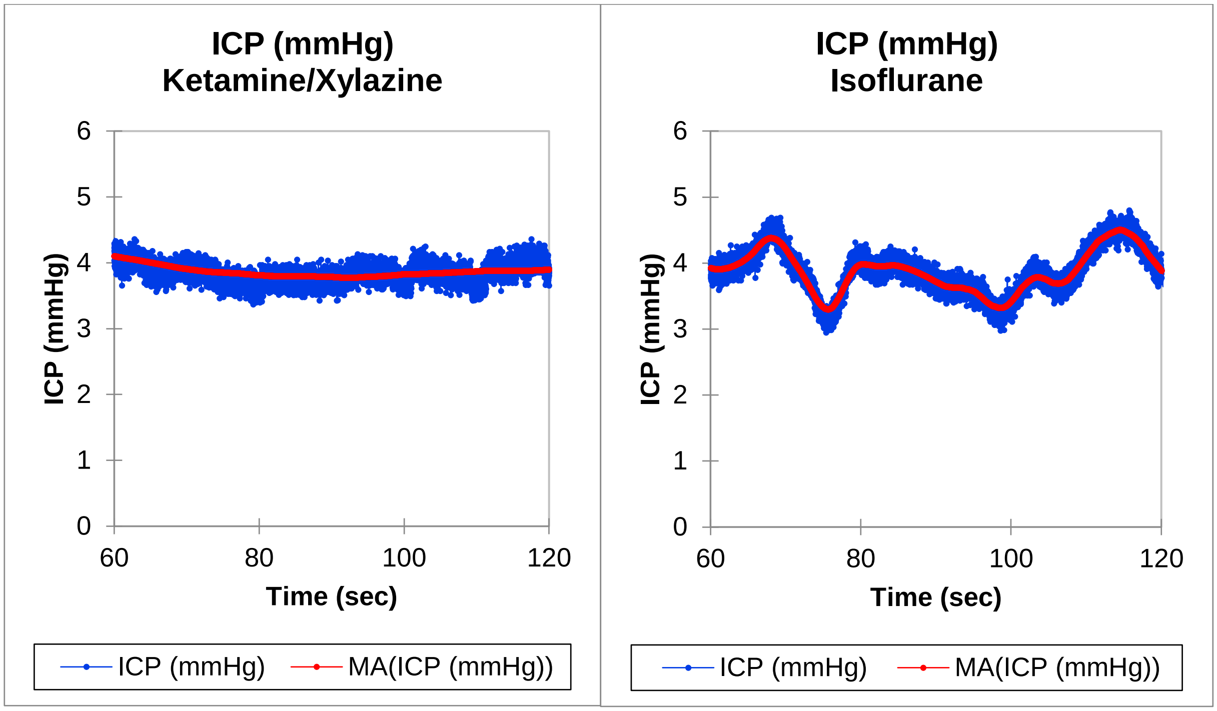

Supplementary Fig. 5*: Intracranial pressure (ICP) measurements conducted via a pressure probe inserted into the cisterna magna in a mouse anesthetized with KX (left) and ISO (right). In both animals the ICP rests at ~ 4 mmHg. Respiratory effort can be observed more prominently in the ISO anesthetized mouse when compared to the KX mouse.*
